# Supplementary material for: Comparative Sensing and Judgment Control System for Temperature Maintenance for Optimal Treatment in Hyperthermic Intraperitoneal Chemotherapy Surgery
Source: Sensors (Basel). 2024 Jan 17;24(2):596. doi: 10.3390/s24020596 (PMC10821041; doi:10.3390/s24020596)
Supplement: Supplementary file 1 [file sensors-24-00596-s001.zip › sensors-2800407-supplementary.pdf]

Algorithm S1. Algorithm coding data.

```
>> % Define input signals
t = 0:0.01:10;  % Time vector
S = [0 0 1 1 0 0 1 1 0 0];  % S input sequence
R = [0 1 0 1 0 1 0 1 0 1];  % R input sequence

% Initialize flip-flop state
Q = zeros(size(t));
Q(1) = 0;  % Initial state of Q

% Iterate over time steps
for i = 2:length(t)
    if S(i) == 0 && R(i) == 0
        % No change
        Q(i) = Q(i-1);
    elseif S(i) == 0 && R(i) == 1
        % Reset
        Q(i) = 0;
    elseif S(i) == 1 && R(i) == 0
        % Set
        Q(i) = 1;
    elseif S(i) == 1 && R(i) == 1
        % Invalid state (both inputs are 1)
        Q(i) = NaN;  % Set output to NaN to indicate
invalid state
    end
end

% Plot the behavior of S-R flip-flop
figure;
plot(t, S, 'b', 'LineWidth', 1.5);
hold on;
plot(t, R, 'r', 'LineWidth', 1.5);
plot(t, Q, 'g', 'LineWidth', 1.5);
ylim([-0.5 1.5]);
xlabel('Time');
ylabel('Signal');
legend('S', 'R', 'Q');
title('Behavior of S-R Flip-Flop');
grid on;
```

In the Figure S1, clock (CK) pulse is applied to RF—flip/flop to induce TC to operate. At this time, pulses corresponding to T_{ref} and T_o were input to TC, resulting in T_D pulses occurring at the output. Therefore, when the TD is input to the LUT, the output of the LUT is T_p, T_s, T_a, and T_x output according to the temperature change as shown in Figure S2, and the operation of the heat exchanger can be induced.

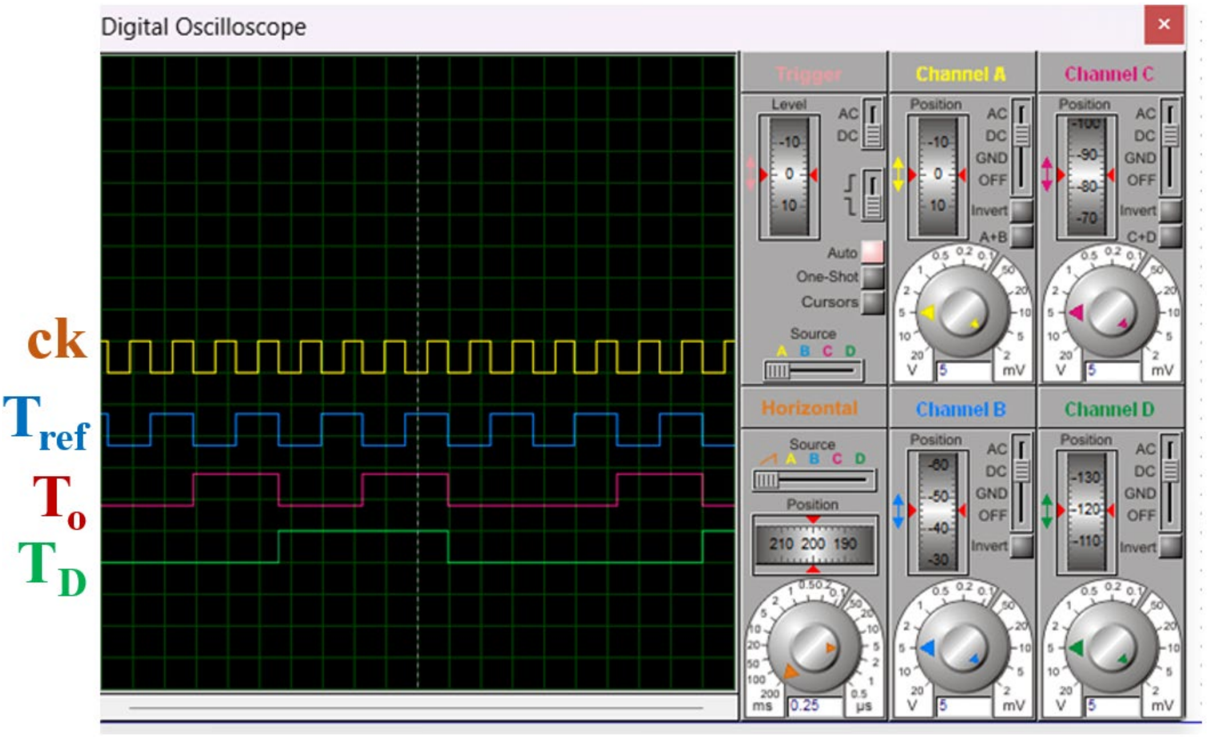

Figure S1. Measurement results for the pulse signal of T_{ref}, T_o, and T_D corresponding to the TC performance.

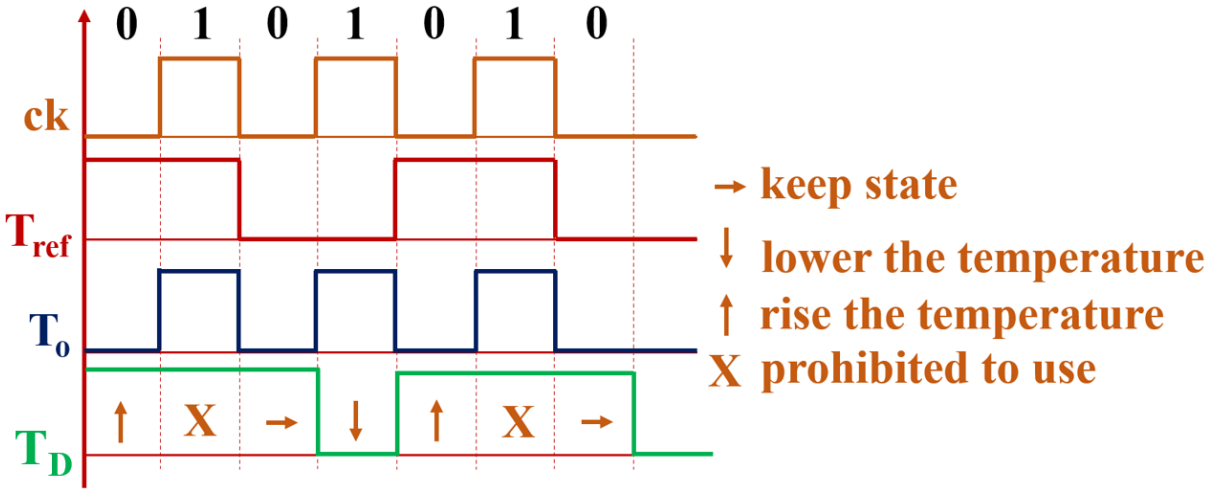

Figure S2. T_D pulse signal output and thermal control concept for comparison/judgment of T_{ref} a nd T_o via TC.
